# Assessment of cognitive disorders in Covid-19 patients in Tunisia

**DOI:** 10.1192/j.eurpsy.2022.1297

**Published:** 2022-09-01

**Authors:** A. Adouni, W. Abdelghaffar, S. Boudriga, B. Somii, H. Blibech, N. Haloui, R. Rafrafi

**Affiliations:** Mongi Slim hospital, Psychiatry, tunis, Tunisia

**Keywords:** cognitive impairment, sequelae, Covid-19

## Abstract

**Introduction:**

Since the beginning of the pandemic, the Sars-cov2 virus remains unclear concerning its clinical manifestations and its sequelae. Few studies have evaluated the existence of cognitive impairment in patients with COVID-19 and estimated its imputability in the development of these disorders.

**Objectives:**

The objectives of this study is to assess cognitive disorders in post-COVID patients.

**Methods:**

A descriptive observational survey was conducted by the psychiatry department of Mongi Slim hospital in Tunis-Tunisia during May-June 2021 among covid-19 patients selected at the first post covid consultation (at 1 month). First, sociodemographic and clinical data were collected, then the evaluation of the cognitive disorders was carried using many scales: MMS (mini mental state), FAB, TMT and the maze task.

**Results:**

Eight patients met the selection criteria with a sex ratio of 6:2 and an average age of 67.5 years (6 with a primary level). The extent of the lesions varied between 10% and 75%. Among the participants, 4 required hospitalization in intensive care: 3 with non-invasive ventilation and 1 needed intubation. The 1-month evaluation found that all the patients had good memory and execution skills with MMS scales >25 and FAB scales >14. Regarding flexibility and planification: 4 of them needed more than 78 seconds to complete the TMT-A ,3 took more than 273 seconds to complete the TMT-B and 2 needed more than 60 seconds to accomplish the maze task (deficient scores).

**Conclusions:**

The screening of cognitive disorders in post-COVID patients is very important for a better management that may require early neurocognitive rehabilitation.

**Disclosure:**

No significant relationships.

